# Machine learning-based prediction of prolonged air leak after uniportal video-assisted thoracic surgery segmentectomy

**DOI:** 10.3389/fonc.2026.1858988

**Published:** 2026-06-19

**Authors:** Liang Chen, Ting Yu, Yanqing Pan, Guodong Ma

**Affiliations:** 1 Department of Thoracic Surgery, Nanjing Chest Hospital, Nanjing, China; 2 Department of Thoracic Surgery, Affiliated Nanjing Brain Hospital, Nanjing Medical University, Nanjing, China; 3 Department of Ophthalmology, Jinling Hospital, Medical School of Nanjing University, Nanjing, China

**Keywords:** machine learning, prolonged air leak, pulmonary, segmentectomy, uniportal video-assisted thoracic surgery

## Abstract

**Objective:**

Prolonged air leak (PAL), defined as air leakage lasting more than 5 days, remains a prevalent complication following uniportal video-assisted thoracic surgery (uVATS) segmentectomy, contributing to extended hospitalization, elevated medical expenditures, and higher postoperative morbidity. This study was designed to develop and internally validate machine learning models for predicting PAL after uVATS segmentectomy using a comprehensive set of clinical, surgical, and physiological variables.

**Methods:**

A retrospective cohort study was conducted including 610 consecutive patients who underwent uVATS segmentectomy between January 2019 and September 2025. Candidate predictors included demographics, comorbidities, pulmonary function, surgical parameters, and tumor characteristics. The dataset was randomly divided into training (70%) and internal test (30%) cohorts. Five machine learning algorithms—logistic regression, elastic net regression, random forest, gradient boosting machine (GBM), and extreme gradient boosting (XGBoost)—were constructed. Model performance was evaluated using the area under the receiver operating characteristic curve (AUC), sensitivity, specificity, positive predictive value (PPV), negative predictive value (NPV), overall accuracy, and decision curve analysis. The Shapley additive explanation (SHAP) algorithm was applied to quantify feature contributions and enhance model interpretability.

**Results:**

A total of 76 (12.46%) patients developed PAL. The XGBoost model achieved the highest discriminative performance, with an AUC of 0.874 [95% confidence interval (CI): 0.833–0.906] in the internal test set, outperforming conventional regression and other machine learning models. SHAP analysis identified five dominant predictive factors: low body mass index (BMI), prolonged operative time, reduced percentage of carbon monoxide lung diffusion capacity (DLCO%), diabetes, and complex segmentectomy.

**Conclusions:**

Machine learning models, especially the XGBoost algorithm, show promising internal performance for predicting PAL following uVATS segmentectomy by integrating multidimensional perioperative variables. The key predictors identified in this study offer evidence-based insights for perioperative risk stratification and individualized care strategies. External multicenter validation, calibration assessment, and prospective clinical evaluation are required before routine clinical implementation.

## Introduction

Anatomical segmentectomy is an accepted intervention for selected patients with early-stage non-small-cell lung cancer (NSCLC) and benign pulmonary lesions, providing comparable oncological efficacy to lobectomy while better preserving postoperative pulmonary function (1, 2). The uniportal video-assisted thoracic surgery (uVATS) approach further enhances the minimally invasive benefits of segmentectomy, reducing postoperative pain, accelerating recovery, and improving cosmetic outcomes, compared with conventional multiportal VATS (3, 4). Despite these advantages, prolonged air leak (PAL) remains one of the most frequent and costly complications after uVATS segmentectomy, with reported incidence ranging from 14.1% to 18.8% (5, 6).

PAL is strongly associated with prolonged chest tube drainage, extended length of hospital stay, increased risk of readmission, and elevated medical expenditures (7). Previous studies have identified several risk factors for PAL, including low body mass index (BMI), impaired pulmonary function, smoking history, complex segmentectomy, and diabetes mellitus (6, 8, 9). However, traditional statistical models fail to capture complex non-linear interactions among high-dimensional clinical variables, limiting their predictive accuracy and clinical utility.

Machine learning algorithms excel in processing multidimensional data and identifying hidden predictive patterns, making them powerful tools for surgical complication prediction (10). Although recent studies have applied machine learning (ML) to predict PAL after lung resection, few have focused specifically on uVATS segmentectomy—a procedure with unique anatomical complexity and technical demands (5, 9, 11). To address this gap, we developed and internally validated a series of machine learning models for PAL prediction after uVATS segmentectomy and used Shapley additive explanation (SHAP) analysis to provide transparent, clinically meaningful interpretability.

## Patients and methods

### Study design and ethical approval

This single-center retrospective study was approved by the Ethics Committee of Nanjing Chest Hospital and performed in accordance with the ethical principles outlined in the 2013 revised Declaration of Helsinki. Informed consent was waived due to the retrospective design and full anonymization of adult patients (age ≥ 18 years).

### Patients

Consecutive patients who underwent elective uVATS anatomical segmentectomy between January 2019 and September 2025 were enrolled.

The inclusion criteria included 1) age ≥ 18 years; 2) complete preoperative, intraoperative, and postoperative data; and 3) pathologically confirmed NSCLC, metastatic lung tumor, or benign pulmonary lesion. The exclusion criteria included 1) conversion to thoracotomy or lobectomy, 2) missing key data, 3) emergency surgery, and 4) pregnancy.

A total of 610 consecutive patients who underwent uVATS segmentectomy between January 2019 and September 2025 were enrolled.

### Surgical procedure

All operations were performed by experienced board-certified thoracic surgeons specializing in uVATS segmentectomy. Patients underwent single-lung ventilation and were placed in the lateral decubitus position. A 3–4-cm incision was created in the fourth or fifth intercostal space along the anterior or midaxillary line, followed by the placement of a wound retractor. A 10-mm 30° thoracoscope and dedicated uVATS instruments were used throughout the procedure. Bronchovascular structures were individually dissected and divided using endoscopic staplers or vascular clips.

The intersegmental plane was identified via the inflation–deflation method or indocyanine green fluorescence. Pulmonary parenchymal division was completed using endoscopic staplers. A standard underwater leak test was performed at the end of the procedure. A single 24-F chest tube was inserted toward the thoracic apex.

### Chest tube management and PAL adjudication

The chest tube removal criteria included no air leakage for ≥24 consecutive hours, drainage <200 mL/24 h, and no significant pneumothorax on chest imaging. No patients were discharged with chest tubes. PAL was retrospectively adjudicated by two independent thoracic surgeons.

### Definitions and variables

PAL was defined as air leakage persisting more than five postoperative days. Segmentectomy was classified into simple and complex subtypes according to the criteria reported by Handa et al. (12). Simple segmentectomies included the resection of the right sixth segment, left sixth segment, left upper division segment, or lingula segment. Complex segmentectomy included segmentectomy other than simple segmentectomy, such as the resection of the right third segment, left ninth segment, right first and third segments, and left ninth and 10th segments.

Candidate predictive variables were selected based on previous literature and clinical relevance, including demographic variables (age, sex, and BMI), comorbidities (hypertension, diabetes, and smoking status), pulmonary function (FEV1% predicted and DLCO% predicted), surgical variables (surgical side, resected lobe, segmentectomy type, additional wedge resection, operative time, and pleural adhesions), and tumor variables (tumor size and histological type).

### Statistical and machine learning analysis

#### Statistical analysis

Continuous variables were tested for normality and reported as mean ± standard deviation or median Iterquartile Range (IQR). Categorical variables were reported as n (%). Comparisons were performed using Student’s t-test, Mann–Whitney U test, or χ^2^ test. Variables with p < 0.10 in univariate analysis were entered into multivariable logistic regression. A two-sided p < 0.05 was considered statistically significant. All analyses were performed using IBM SPSS 23.0 and Python 3.12.13.

#### Machine learning models

Five algorithms were used: logistic regression, elastic net regression, random forest, gradient boosting machine (GBM), and extreme gradient boosting (XGBoost). The cohort was randomly split at a 7:3 ratio for training and internal testing. Class imbalance was addressed using class weighting. A 10-fold cross-validation was used for hyperparameter tuning with early stopping to avoid overfitting. Preprocessing included standard scaling for continuous variables; no missing data were present.

Model performance was evaluated using the area under the receiver operating characteristic curve (AUC), accuracy, sensitivity, specificity, positive predictive value (PPV), negative predictive value (NPV), and decision curve analysis. The DeLong test was used to compare AUCs between models. The internal test set was used only once after model selection.

SHAP analysis was applied to the XGBoost model to quantify feature contributions. Mean absolute SHAP values were used to rank importance. SHAP values reflect model-specific contributions rather than independent epidemiological associations.

## Results

### Baseline characteristics of the total cohort

Among 610 patients, 76 (12.46%) developed PAL. The PAL prevalence was 12.41% in the training set and 12.57% in the internal test set. The mean age was 64.92 ± 7.82 years, and 358 (58.69%) were male. The mean BMI was 23.99 ± 2.77 kg/m^2^. A total of 538 (88.20%) patients had primary NSCLC, and 397 (65.08%) underwent complex segmentectomy. The baseline characteristics are summarized in **Table 1**.

**Table 1:** Baseline characteristics of patients with and without PAL.

Variable	Total (n = 610)	No PAL (n = 534)	PAL (n = 76)	P-value
Age, years (mean SD)	64.92 (7.82)	64.77 (7.79)	65.92 (8.07)	0.233
Male gender (n, %)	358 (58.69)	320 (59.93)	38 (50)	0.107
BMI, kg/m^2^ (mean, SD)	23.99 (2.77)	24.13 (2.74)	22.99 (2.79)	0.001
Smoking status (n, %)				0.748
Current smoker	92 (15.08)	80 (14.98)	12 (15.79)	
Ex-smoker	210 (34.43)	183 (34.27)	27 (35.53)	
Never smoker	308 (50.49)	271 (50.75)	37 (48.68)	
Hypertension (n, %)	206 (33.77)	181 (33.90)	25 (32.89)	0.898
Diabetes (n, %)	89 (14.6)	72 (13.48)	19 (25)	0.015
FEV1% (mean, SD)	86.96 (11.13)	87.23 (11.35)	85.15 (9.24)	0.128
DLCO% (mean, SD)	82.60 (12.47)	83.10 (12.76)	78.45 (9.62)	0.002
Location of tumor (upper lobe) (n, %)	283 (46.39)	243 (45.51)	40 (52.63)	0.269
Side of operation (left) (n, %)	311 (50.98)	276 (51.69)	35 (46.05)	0.392
Complex segmentectomies (n, %)	397 (65.08)	338 (63.30)	59 (77.63)	0.014
Additional wedge resection (n, %)	80 (13.11)	67 (12.55)	13 (17.11)	0.277
Pleural adhesions (n, %)	81 (13.28)	72 (13.48)	9 (11.84)	0.859
Tumor size, cm (mean, SD)	1.65 (0.38)	1.65 (0.38)	1.68 (0.38)	0.520
Histology (n, %)				0.510
Benign	55 (9.02)	46 (8.61)	9 (11.84)	
Primary NSCLC	538 (88.20)	474 (88.10)	64 (84.21)	
Metastasis	17 (2.79)	14 (2.62)	3 (3.95)	
Operative time, min (mean, SD)	111.69(22.10)	110.67 (22.29)	118.83 (19.37)	0.003

SD, standard deviation; BMI, body mass index; FEV1%, forced expiratory volume in 1 second as a percentage of predicted value; DLCO%, diffusing capacity of the lungs for carbon monoxide as a percentage of predicted value; NSCLC, non-small-cell lung cancer; PAL, prolonged air leak (>5 days).

### Univariable and multivariable logistic regression

Univariate analysis identified five variables associated with PAL: BMI, diabetes, DLCO%, complex segmentectomy, and operative time (all p < 0.05). Multivariable logistic regression confirmed these variables as independent risk factors (**Table 2**). For continuous variables, Odds Rations (ORs) correspond to a per 1 kg/m^2^ increase in BMI, a per 1% increase in DLCO%, and a per 1-minute increase in operative time.

**Table 2:** Multivariable logistic regression for predictors of prolonged air leak.

Variable	OR (95% CI, p)	P-value
BMI	0.849 (0.770–0.935)	0.001
DLCO%	0.961 (0.934–0.988)	0.005
Operative time	1.018 (1.006–1.031)	0.004
Diabetes	2.228 (1.199–4.142)	0.011
Complex segmentectomy	2.132 (1.171–3.879)	0.013

BMI, body mass index; DLCO%, percentage of carbon monoxide lung diffusion capacity.

### Machine learning model performance

To ensure methodological rigor and clinical interpretability, a multivariate logistic regression model was first established as a reference. This baseline model helped clarify the direction and magnitude of variable effects. On this basis, we systematically compared different machine learning algorithms to evaluate their incremental value in modeling non-linear relationships and feature interactions.

**Figure 1:**
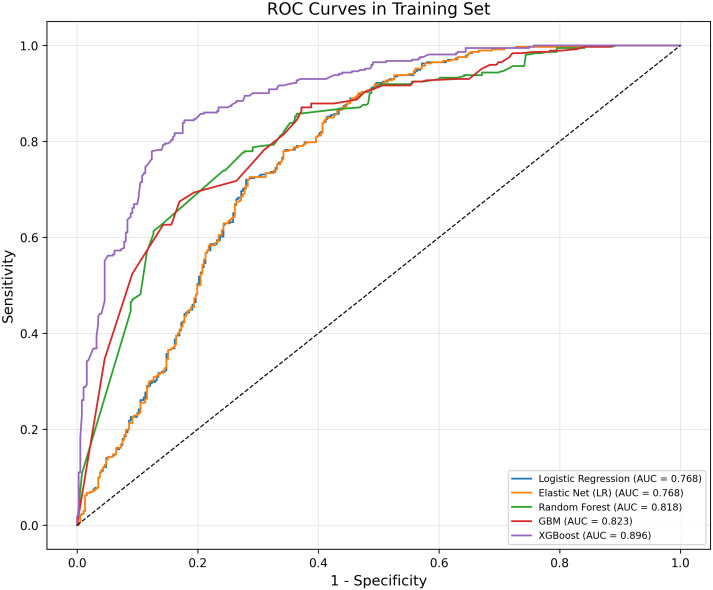
Receiver operating characteristic (ROC) curves of machine learning models in the training cohort. The extreme gradient boosting (XGBoost) model shows the highest discriminative performance.

**Figure 2:**
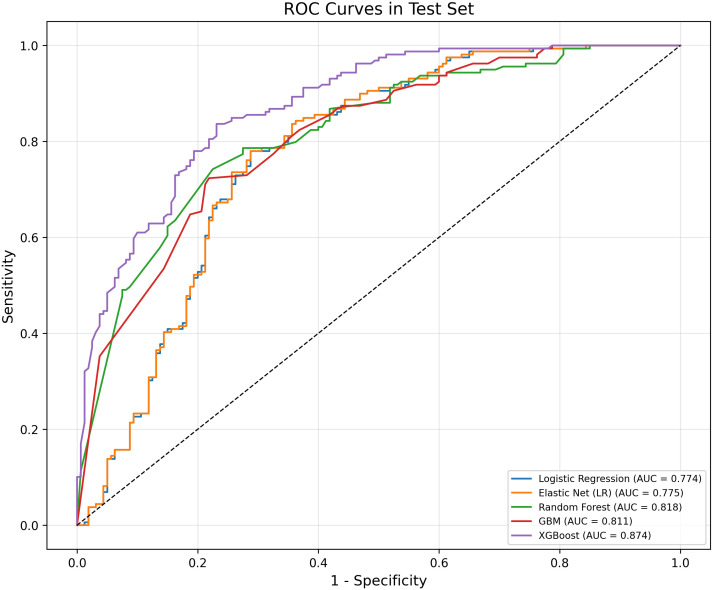
Receiver operating characteristic (ROC) curves of machine learning models in the internal test cohort. The extreme gradient boosting (XGBoost) model achieves the highest area under the receiver operating characteristic curve (AUC).

The XGBoost algorithm achieved the best performance in both training and internal test sets, with an AUC of 0.874 [95% confidence interval (CI) 0.833–0.906] in the test set. The DeLong test confirmed significantly higher discrimination than logistic regression (p < 0.05). Calibration was satisfactory (slope = 0.89, intercept = 0.12; Brier score = 0.082). Decision curve analysis showed positive net benefit in clinically relevant threshold ranges. Therefore, XGBoost was selected as the optimal predictive model. The detailed performance metrics are presented in **Table 3** and **Figures 1**, **2**.

**Table 3:** Performance of machine learning models in the training set and test set.

Model	AUC (95% CI)	Sensitivity (%)	Specificity (%)	PPV (%)	NPV (%)	Accuracy (%)
Logistic regression						
Training	0.768 (0.733–0.802)	75	66.8	69.4	72.7	70.9
Testing	0.774 (0.721–0.826)	77.4	71.2	72.8	76	74.3
Elastic net regression						
Training	0.768 (0.733–0.802)	75	66.8	69.4	72.7	70.9
Testing	0.770 (0.722–0.827)	77.4	71.2	72.8	76	74.3
Random forest						
Training	0.818 (0.790–0.849)	85.2	63.9	70.3	81.2	74.6
Testing	0.818 (0.772–0.862)	86.2	58.1	67.2	80.9	72.1
GBM						
Training	0.823 (0.794–0.850)	87.1	61.2	69.2	82.5	74.2
Testing	0.811 (0.762–0.855)	87.4	55	65.9	81.5	71.2
XGBoost						
Training	0.896 (0.874–0.918)	86.3	76.8	78.9	84.8	81.6
Testing	0.874 (0.833–0.906)	85.5	70	73.9	83	77.7

AUC, area under the receiver operating characteristic curve; CI, confidence interval; PPV, positive predictive value; NPV, negative predictive value; GBM, gradient boosting machine; XGBoost, extreme gradient boosting.

### Feature importance and interpretability

**Figure 3:**
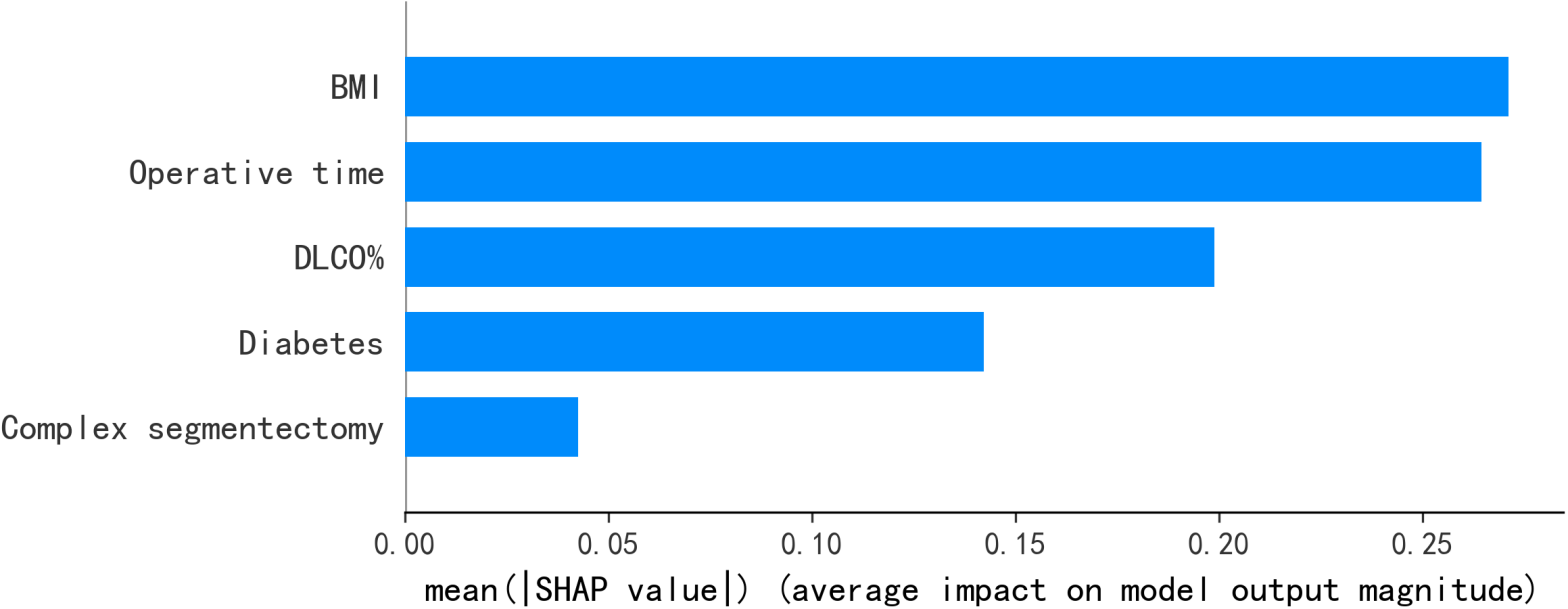
Feature importance ranked by mean absolute Shapley additive explanation (SHAP) values for the optimal extreme gradient boosting (XGBoost) model. The top five predictors of prolonged air leak (PAL) are low body mass index (BMI), prolonged operative time, reduced percentage of carbon monoxide lung diffusion capacity (DLCO%), diabetes, and complex segmentectomy.

**Figure 4:**
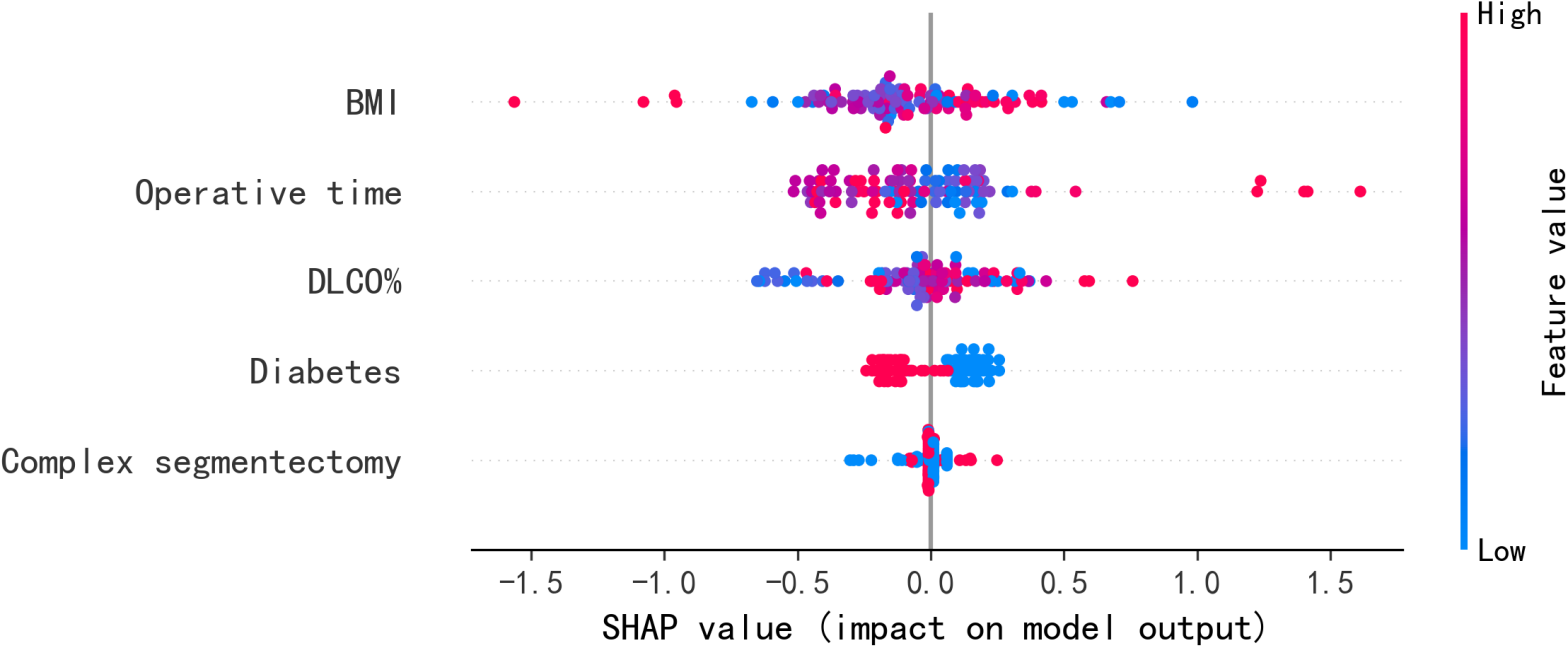
Shapley additive explanation (SHAP) summary plot for key predictors of prolonged air leak (PAL).

To improve the clinical interpretability of the XGBoost model, SHAP analysis was used to quantify and visualize each feature’s contribution to PAL prediction. Mean absolute SHAP values were calculated to assess relative variable importance, with higher values indicating greater influence on model output. SHAP analysis revealed consistent top five predictors—low BMI, prolonged operative time, reduced DLCO%, diabetes, and complex segmentectomy—all of which were associated with increased PAL risk (**Figure 3**). The SHAP summary plot (**Figure 4**) illustrates the distribution and directional effects of the candidate features: positive SHAP values indicate increased PAL risk, while negative values indicate reduced risk. The color gradient represents feature magnitude (red = high value and blue = low value), and point density reflects sample size.

## Discussion

PAL remains one of the most common and clinically challenging complications following uVATS segmentectomy, directly contributing to prolonged chest tube drainage, extended length of hospital stay, increased rates of hospital readmission, and substantial incremental healthcare expenditure (7, 11, 13). In this study of 610 patients, we developed and internally validated a series of machine learning models for the perioperative prediction of PAL. The XGBoost model achieved excellent discriminative performance (AUC = 0.874), and SHAP analysis identified low BMI, prolonged operative time, reduced DLCO%, diabetes, and complex segmentectomy as the most influential predictive factors. These findings provide a promising tool for perioperative risk stratification and personalized perioperative care.

Low BMI was the strongest predictive factor for PAL, consistent with recent large-cohort studies (6, 8, 11). Malnutrition impairs collagen synthesis, fibroblast proliferation, and tissue repair—key processes for healing parenchymal defects after segmentectomy. Patients with low BMI often have insufficient lean body mass and hypoalbuminemia, which compromise postoperative tissue healing. However, albumin was not measured in this study, and such mechanistic interpretations remain hypothetical. Our results support preoperative nutritional screening and targeted supplementation for underweight patients to reduce PAL risk.

Reduced DLCO% was another major predictor, reflecting impaired alveolar–capillary membrane integrity and diminished parenchymal healing capacity (6, 8, 9). Lower DLCO% correlates with emphysema, interstitial lung disease, and chronic obstructive pulmonary disease, all of which increase lung fragility and air leak persistence. Gioutsos et al. confirmed that DLCO% < 70% is strongly associated with PAL after uVATS segmentectomy (8). Our data extend these findings by demonstrating a continuous dose–response relationship between decreasing DLCO% and increasing PAL risk, supporting routine DLCO% measurement in preoperative risk assessment.

Surgical complexity, represented by complex segmentectomy and prolonged operative time, was independently associated with PAL. Complex segmentectomies involve extensive dissection of intersegmental planes and greater parenchymal manipulation, increasing tissue trauma and air leak risk (12). Prolonged operative time exacerbates inflammation and ischemia, further delaying healing (8, 9). These findings highlight the importance of surgical standardization, surgeon experience, and tissue-sparing techniques to minimize PAL in complex cases.

Diabetes mellitus was confirmed as an independent risk factor for PAL. Chronic hyperglycemia induces microvascular dysfunction, impairs immune function, and delays tissue repair, approximately doubling the risk of PAL after lung resection (11). Our multivariable model validates this association in a uVATS-specific cohort. Preoperative optimization of glycemic control, including Hemoglobin A1c (HbA1c) evaluation and personalized management, is critical in reducing PAL in diabetic patients.

The superior performance of the XGBoost model relative to conventional logistic regression highlights the incremental value of ML in capturing non-linear relationships and high-dimensional interactions among predictive variables (14). Traditional regression models remain limited in their ability to resolve complex interactions between nutritional status, pulmonary function, and surgical complexity, whereas tree-based ensemble methods such as XGBoost excel in modeling these relationships (10). To our knowledge, this study reports one of the highest AUC values (0.874) for PAL prediction, specifically in uVATS segmentectomy. The integration of SHAP analysis addresses the “black-box” limitation of ML models, providing transparent, patient-specific interpretability essential for clinical adoption (15). However, SHAP values reflect model-specific contributions rather than independent epidemiological effects.

Our findings are consistent with those of Gioutsos et al. (8), who identified low BMI, reduced DLCO%, complex segmentectomy, and diabetes as key predictors of PAL after uVATS segmentectomy. The present study extends those findings using machine learning to model non-linear relationships; however, the incremental clinical value over conventional regression requires prospective evaluation.

This study has several limitations. First, the single-center retrospective design may introduce selection bias. Second, only internal validation was performed; external multicenter or temporal validation is required to confirm generalizability. Third, a single random split may introduce model instability. Fourth, class imbalance was addressed using class weighting rather than resampling. Fifth, radiomic and molecular biomarkers were not included, but they may improve predictive performance in future studies. Finally, SHAP importance may be affected by feature correlation and sample size.

## Conclusions

The XGBoost machine learning model shows promising internal performance for predicting PAL after uVATS segmentectomy using routinely available perioperative variables. The five key risk factors—low BMI, prolonged operative time, reduced DLCO%, diabetes, and complex segmentectomy—are pathophysiologically coherent and clinically informative. This model is not yet ready for routine clinical implementation. External multicenter validation, calibration assessment, and prospective interventional studies are required to confirm generalizability and real-world utility before clinical use.

## Data Availability

The raw data supporting the conclusions of this article will be made available by the authors, without undue reservation.
